# Supplementary material for: What do Brazilian owners know about canine obesity and what risks does this knowledge generate?
Source: PLoS One. 2020 Sep 21;15(9):e0238771. doi: 10.1371/journal.pone.0238771 (PMC7505417; doi:10.1371/journal.pone.0238771)
Supplement: S1 Appendix — (DOCX) [file pone.0238771.s001.docx]

**What do Brazilian owners know about canine obesity and what risks does this knowledge generate?**

Supporting information

**S1 Appendix**

Questionnaire used to determine the opinions of 966 dog owners about obesity and weight management.

| \| **– Your age** \| \| --- \| \| a) Between 18 and 24 years \| \| b) Between 25 and 34 years \| \| c) Between 35 and 44 years \| \| d) Between 45 and 59 years \| \| e) Between 60 and 75 years \| \| f) Older than 76 years \| \|  \| \| **- Education** \| \| a) From 1st to 4th grade \| \| b) From 5th to 8th grade \| \| c) High School \| \| d) College/university \| \| e) Specialization \| \| f) Did not study \| \|  \| \| **– Adding your income with the income with all the people that live with you, how much is your mean Family monthly income?** \| \| a)     No income \| \| \| b)     Up to U$79,31 \| \| \| c)     From U$158,63 to U$284,94 \| \| \| d)   From U$285,19 to U$997,80 \| \| \| e)   From U$998,04 to U$ 2.428,40 \| \| \| f)   Above U$ 2.428,40 \| \| \|  \| \| **- What is your current job?** \| \| a) Government (public sector) \| \| b) Company (private or state) \| \| c) Non-governmental organization \| \| d) Autonomous \| \| e) Rural property \| \| f) Unemployed \| \| g) Retired \|   **– Do you believe that treats influence weight gain?**  Yes ( ) No ( )  **– In your opinion, an obese dog is more limited (has more difficulty) to play, run, walk and feels more heat than a dog with ideal weight?** Yes ( ) No ( )  **- In case your dog is overweight, would you take part in a weight loss program?**  Yes ( ) No ( )  **-Do you believe that obesity can present risks to the animal’s health?**  Yes ( ) No ( )  **– Do you think a trained professional is needed for the weight loss program?**  Yes ( ) No ( )  **– Where would search for help regarding weight loss program if your dog was obese?**   1. Pet food industry 2. Pharmaceutical company 3. Dog trainers 4. Internet 5. Veterinary clinic 6. Dog breeder 7. Friends, family and/or neighbors   **- What would you do to help your dog lose weight? (Mark as many as necessary)**   1. Natural products for weight loss 2. Commercial supplements for weight loss 3. Exercise 4. Hypocaloric pet food 5. Use of drugs/medicine 6. Reduce treats 7. Not offering treats 8. Homemade diet   **– What are the difficulties that you believe may surface during your dog’s weight loss program?**  **-**Cost/price of pet food? Yes ( ) No ( )  -Frequent exercise? Yes ( ) No ( )  -Change of feeding habits? Yes ( ) No ( )  -All your family members following instructions? Yes ( ) No ( )  -The animal being hungry? Yes ( ) No ( )  -Not resisting the temptation of giving treats? Yes ( ) No ( )  -The animal will suffer from the treatment? Yes ( ) No ( )  -The animal is too old for treatment? Yes ( ) No ( )  -The animal will treat you like you less if take out treats? Yes ( ) No ( )  **-Evaluation of owner’s perception regarding their dog’s body condition score**  ( ) Underweight ( )Ideal ( )Overweight ( )Obese |
| --- | --- | --- | --- | --- | --- | --- | --- | --- | --- | --- | --- | --- | --- | --- | --- | --- | --- | --- | --- | --- | --- | --- | --- | --- | --- | --- | --- | --- | --- | --- | --- | --- | --- | --- | --- | --- | --- | --- |

| **– Idade**   1. Entre 18 e 24 anos 2. Entre 25 e 34 anos 3. Entre 35 e 44 anos 4. Entre 45 e 59 anos 5. Entre 60 e 75 anos 6. Acima de 76 anos   **- Escolaridade**   1. Da 1ª à 4ª série do Ensino Fundamental (antigo primário) 2. Da 5ª à 8ª série do Ensino Fundamental (antigo ginásio) 3. Ensino Médio (antigo 2º grau) 4. Ensino Superior 5. Especialização 6. Não estudou     **– Somando a sua renda com a renda das pessoas que moram com você, quanto é, aproximadamente, a renda familiar mensal? (Marque apenas uma resposta)**   1. Nenhuma renda. 2. Até R$ 324 3. De R$ 648,00 até R$ R$ 1.164 4. De R$ 1.165 a R$ 4.076 5. De R$ 4.077 a R$ 9.920 6. ACIMA R$ 9.921   **- Em que você trabalha atualmente?**   1. Governo (Setor Público) 2. Empresa (privada ou estatal) 3. ONG 4. Conta própria 5. Propriedade Rural 6. Desempregado 7. Aposentado   **- Você acredita que os petiscos influenciam o ganho de peso?**  Sim ( ) Não ( )  **- Na sua opinião, um cão obeso pode ser mais limitado, (tem mais dificuldade) para brincar, correr, caminhar e sente mais calor que um cão de peso ideal?**  Sim ( ) Não ( )  **- Caso seu animal apresente-se acima do peso, você aceitaria participar de um tratamento para perda de peso?**  Sim ( ) Não ( )  **- Você acredita que a obesidade possa oferecer riscos à saúde do animal?**  Sim ( ) Não ( )  **- Você acha necessário um profissional habilitado para ajudar o animal perder?**  Sim ( ) Não ( )  **- Onde você buscaria ajuda para perda de peso do seu cão caso ele fosse obeso?**   1. Indústria pet food 2. Companhia farmacêutica 3. Adestradores 4. Internet 5. Clínica veterinária 6. Criador 7. Amigos, vizinho, familiars   **- O que você faria para seu animal perder peso? (Marque quantas julgar necessário)**   1. Produtos naturais para perder peso 2. Suplementos alimentares para perda de peso (comercial) 3. Praticar exercícios 4. Ração hipocalórica (menor caloria) 5. Uso de medicamentos 6. Diminuir petiscos 7. Não oferecer petiscos 8. Dieta caseira   **- Quais as dificuldades que você acredita que possam surgir durante a perda de peso do seu animal:**  - Custo/preço da ração? Sim ( ) Não ( )  - Realização de exercícios frequentes? Sim ( ) Não ( )  - Mudança de hábitos alimentares? Sim ( ) Não ( )  - Todos membros da família seguirem as recomendações Sim ( ) Não ( )  - O animal sentir fome? Sim ( ) Não ( )  - Não resistir a tentação de fornecer petiscos ao animal? Sim ( ) Não ( )  - Acredita que o animal sofrerá com o tratamento? Sim ( ) Não ( )  - Acredita que o animal está idoso demais para aderir ao tratamento? Sim ( ) Não ( )  - Acredita que o animal se tornará menos carinhoso por retirar os petiscos? Sim ( ) Não ( )  **-Avaliação da percepção do proprietário em relação ao escore de condição corporal do cão:**  ( ) magro ( ) Ideal ( ) sobrepeso ( ) obeso |
| --- |
